# Virulence of methicillin-resistant *Staphylococcus aureus* modulated by the YycFG two-component pathway in a rat model of osteomyelitis

**DOI:** 10.1186/s13018-019-1508-z

**Published:** 2019-12-12

**Authors:** Shizhou Wu, Yunjie Liu, Lei Lei, Hui Zhang

**Affiliations:** 1 Department of Orthopedics, West China Hospital, Sichuan University, No.37 Guoxue Alley, Chengdu City, 610041 Sichuan China; 2 West China School of Public Health, Sichuan University, Chengdu, China; 3 State Key Laboratory of Oral Diseases, Department of Preventive Dentistry, West China Hospital of Stomatology, Sichuan University, NO.14 Renmin South Road, Chengdu City, 610041 Sichuan China

**Keywords:** *Staphylococcus aureus*, Antisense RNA, YycFG two-component regulatory system, MRSA, Biofilm

## Abstract

**Objectives:**

Methicillin-resistant *Staphylococcus aureus* (MRSA) strains present an urgent medical problem in osteomyelitis cases. Our previous study indicated that the YycFG two-component regulatory pathway is associated with the bacterial biofilm organization of MRSA strains. The aim of this study was to investigate the regulatory roles of AS*yycG* in the bacterial biofilm formation and the pathogenicity of MRSA strains using an antisense RNA strategy.

**Methods:**

An AS*yycG*-overexpressing MRSA clinical isolate was constructed. The bacterial growth was monitored, and the biofilm biomass on bone specimens was examined using scanning electron microscopy and confocal laser scanning microscopy. Furthermore, quantitative RT-PCR (QRT-PCR) analysis was used to measure the expression of *yycF/G/H* and *icaA/D* in the MRSA and AS*yycG* strains. The expression of the YycG protein was quantified by Western blot assays. We validated the role of AS*yycG* in the invasive ability and pathogenicity of the strains in vivo using histology and peptide nucleic acid fluorescent in situ hybridization.

**Results:**

The results showed that overexpression of AS*yycG* lead to a reduction in biofilm formation and exopolysaccharide (EPS) synthesis compared to the control MRSA strains. The AS*yycG* strains exhibited decreased expression of the *yycF/G/H* and *icaA/D* genes. Furthermore, Western blot data showed that the production of the YycG protein was inhibited in the AS*yycG* strains. In addition, we demonstrated that AS*yycG* suppressed the invasive ability and pathogenicity of the strain in vivo using an SPF (specific pathogen free) rat model.

**Conclusion:**

In summary, the overexpression of AS*yycG* leads to a reduction in biofilm formation and bacterial pathogenicity in vivo, which provides a potential target for the management of MRSA-induced osteomyelitis.

## Introduction

Osteomyelitis is one type of severe bone infection, and open fractures of long bones are the most frequent etiology [1]. Among the various types of open long bones fractures, tibia fractures have the highest rate of infection due to the lack of soft tissue coverage [2]. The incidence of infection after fracture fixation may be more than 30% in complex open tibia fractures [3]. Surgical interventions are required in aggressive surgical debridement and reconstruction and antibiotic treatments are part of current osteomyelitis management approaches [4]. *Staphylococcus aureus* (*S. aureus*) is the most common isolate in polymicrobial infections [5]. *S. aureus*, a Gram-positive opportunistic pathogen, is widely found in the human skin, nares, and gastrointestinal tracts [6]. As the incidence of methicillin-resistant *S. aureus* (MRSA) is increasing [4], the management of osteomyelitis is of growing concern. In cases of very serious infection, the surgical consequences include radical debridement, resulting in bone or soft-tissue defects and amputation or establishment of a continuous fistula, which may be the only treatment alternative [7]**.** MRSA has recently been listed by the World Health Organization as one of the priority pathogens threatening human health [8]. An extended hospital stay and excessive antibiotic therapy are required in patients with a definite diagnosis of MRSA infection; however, these patients have 2-fold higher mortality rates than noninfected patients [9]. In the USA, the annual cost of treating MRSA infections was documented to be between $3.2 billion and $4.2 billion [10].

Two-component regulatory systems (TCSs) are elements that are essential for bacterial adaption to various environmental changes [11]. However, only a few TCSs are vital for bacterial survival [12]. Among the seventeen encoded TCSs identified in *S. aureus*, YycFG is the only TCS essential for bacterial viability, which means that the deletion of this system by homologous recombination results in bacterial death. The YycFG TCS plays a crucial role in cellular structure, physiology, and biofilm organization [13]. Biofilms are formed by communities that colonize and grow on a self-produced extracellular polymeric substance [14]. The matrix of the three-dimensional structured *S. aureus* biofilm is mainly composed of a polysaccharide intercellular adhesin (PIA) encoded by *icaADBC* [15]. It has been reported that the increased expression of *icaB* may have contributed to the pathogenicity of *S. aureus* [6]. Although previous results showed a potential association between YycFG and MRSA strain virulence in vitro [16], little is known about the effects of the YycFG pathways on MRSA strains in vivo.

Since YycFG is an essential TCS for maintaining cell viability, using homologous recombination to create a gene deletion mutant was not successful [13]. A single-structured antisense RNA (asRNA) can interact with a complementary messenger RNA (mRNA), resulting in the inhibition of the translation of a functional protein. To further investigate the functions of YycFG, we used antisense RNA (asRNA) to interfere with *yycG* gene expression in MRSA (AS*yycG*) strains by stimulating sequence-specific mRNA degradation [17]. In this study, we used MRSA and AS*yycG* strains to inoculate the tibia bone tissue in a rat model to investigate the pathogenicity and invasive ability of *S. aureus*. The morphological, histological, and immunological properties of rats with osteomyelitis were also discussed. We hypothesized the YycFG TCS regulates the pathogenesis of MRSA in vivo and could be a potential effective therapeutic target for the clinical management of MRSA osteomyelitis.

## Methods

### Bacterial strains and growth conditions

The bacterial strains of MRSA were isolated from a patient with chronic osteomyelitis. The strains were kindly provided from the Affiliated Hospital Experimental Department. Pure growth of a single colony was achieved on conventional Baird-Parker (BP) agar. The ATCC29213 strain was one of the common sensitive reference strains [16]. *S. aureus* strains were cultured on tryptic soy agar (TSA) or in TSB broth (Oxoid, Basingstoke, UK) supplemented with 0.5% glucose. The strains were cultured to mid-exponential phase (optical density at 600 nm [OD_600_] = 0.5) in TSB medium for further research. To propagate the MRSA strains, 50 μL of mid-log-phase cells were inoculated in triplicate into 1 mL TSB broth supplemented with 0.5% glucose.

### Construction of the AS*yycG* mutants

The shuttle plasmid pDL278 was used to express the antisense *yycG* (AS*yycG*) sequence. An AS*yycG* overexpression MRSA clinical strain (AS*yycG* mutant) was constructed as previously described with some modifications [18]. First, the AS*yycG* sequences and the promoter sequences were synthesized (Sangon Biotech, Shanghai, China). Next, the antisense sequences were ligated into the pDL278 vector at the BamHI and EcoRI restriction sites, generating the recombinant plasmid pDL278 AS*yycG* (AS*yycG* fragment). This plasmid was then transferred into the MRSA isolates. For the transformation, MRSA strains were grown to mid-exponential phase, and the competence stimulating peptide (CSP) was added to the culture at a final concentration of 1 μg/mL. Simultaneously, recombinant pDL278 AS*yycG* was added and incubated for 60 min. The AS*yycG* strains were isolated using TSB plates that contained 500 μg/mL of spectinomycin for selection. MRSA strains containing the pDL278 empty plasmid were used as a control.

### Biofilm formation and crystal violet microtiter assay for biofilm biomass determination

A pure single colony of *S. aureus* was selected from the TSB agar plates. Then, the colony was further cultured in TSB medium to mid-exponential phase (optical density at 600 nm; OD_600_ 0.5). Sterile glass slides were placed in 24-well polystyrene culture plates, and the biofilm was established for 24 h. The biomass of the *S. aureus* biofilms was assessed by crystal violet (CV) assay as previously described [13]. The biofilms obtained after 24 h of culture in TSB medium were dried in air and stained with 0.1% (w/v) crystal violet for 15 min at room temperature. The bound dye from the stained biofilm cells was solubilized with destaining solution (8:2 ethanol: acetone). The destaining solution was then transferred into a new 96-well plate, and the biofilm biomass was quantified by measuring the OD value at 550 nm.

### Protein extraction and western blotting of the in vitro biofilm

The total proteins of *S. aureus* biofilm cells were extracted and resuspended in phosphate-buffered saline (PBS, pH 7.3). The cells were mechanically disrupted using a FASTPREP Beater apparatus (MP Biomedicals, Irvine, CA) with glass beads (diameter 0.1 mm) for three cycles of 20 s followed by 60 s rest on ice. The supernatants (100 μL) were cleared by centrifugation (14,000×*g*, 1 min, 4 °C), and the protein concentration was determined by Bradford assay (BioRad) according to the manufacturer’s instructions. Equal amounts of protein (20 μg) were mixed with Laemmli sample buffer (Bio-Rad) in boiling water for 10 min and loaded on a precast 4–20% gradient gel (Bio-Rad). The proteins were separated and then semidry electrotransferred to PVDF membranes (Thermo Scientific). Polyclonal antibodies against r-YycG were produced using the standard 70-day rabbit protocol (AbMax Biotechnology Co., Ltd. Beijing, China). The membranes were blocked in 5% (w/v) nonfat dry milk at room temperature for 2 h and then probed with the purified YycG -specific rabbit antibodies diluted 1:1000. The membranes were then washed and incubated with goat anti-rabbit secondary antibodies conjugated with horse-radish peroxidase (dilution 1:10,000). The protein signals were detected with the Immobilon Western Chemiluminescent HRP substrate kit (Millipore). A Bio-Rad GS-700 Imaging Densitometer was used to determine the signal densities of the Western blot bands.

### Bone specimen preparation and biofilm assessment in the in vitro bone samples

The anteromedial tibia cortex of the healthy rats were split longitudinally and sliced (4 × 4 mm) using a hard-tissue cutting machine (Buehler, Chicago, IL, USA). The bone specimen was cleaned ultrasonically in distilled water for 10 min, stored in 10 mM phosphate-buffered saline (PBS, pH 7.0) at 4 °C, and used within 1 week. Then, 1 mL of the mid-exponential phase of the *S. aureus* suspension was placed on the specimens. After anaerobic inoculation for 24 h at 37 °C, the bone specimens were washed twice with PBS to remove the supernatants. To assess the formed biomass, the exopolysaccharides (EPS) matrix of the *S. aureus* biofilms was stained with Alexa 647-labeled dextran conjugate (Invitrogen, Eugene, OR, USA), and the bacterial cells in the biofilm were labeled with SYTO9 (Invitrogen, Carlsbad, CA, USA). Then, confocal laser scanning microscopy was performed using a microscope (CLSM, TSP SP2; Leica, Solms, Germany). For scanning electron microscopy (SEM), the samples were washed twice with PBS buffer and fixed with 2.5% glutaraldehyde. The cells were then serially dehydrated with increasing concentrations of ethanol (30%, 50%, 70%, 80%, 95%, and 100%), dried with liquid CO_2_ to critical point, and coated with gold powder. The sessile biofilms were then observed with a scanning electron microscope (Inspect Hillsboro, OR, USA).

### Animal model of osteomyelitis

Animal experiments were approved by the institutional Animal Welfare Committee. Female Sprague-Dawley rats (260–280 g) were chosen for the animal experiments, and all procedures were conducted as previously described [19]. The animals were anesthetized using ketamine and xylazine. The hind legs were shaved and disinfected with poly (vinylpyrrolidine)-iodine. The anteromedial tibia cortex of the rats was exposed through a longitudinal incision of 1 cm in length. A hole with 0.1 cm diameter was made with a high-speed drill to expose the medullary cavity. Ten animals were divided into two groups, including a group (*n* = 5) injected with 40 μl of the mid-exponential phase MRSA suspension and a group (*n* = 5) inoculated with 40 μl of the mid-exponential phase AS*yycG* suspension. Then, the wound was sutured. Four weeks post-operation, the animals were sacrificed, and bone specimens were obtained for further analysis.

### Micro-CT imaging in vivo

3D images of the rat tibias were taken using the Quantum GX Micro-CT System (PerkinElmer, Waltham, MA) as previously described [20]. The rats were sedated using an isoflurane (1–5%) and oxygen (2 L/min) mixture during the imaging procedures and placed in the supine position inside the cabinet. The scanning conditions were used as follows: kV = 90, CT μA = 72, and 360° scan time = 8 s. We analyzed the three-dimensional reconstruction images with Analyze 12.0 (PerkinElmer, Waltham, MA). The bone density around the infective sites was calculated by the percent bone volume (BV) divided by total volume (TV, %).

### Histological evaluations and scanning electron microscopy of the tibia shaft

The rat tibia specimens were prepared for SEM and for histological evaluations as previously described [21]. The rats were euthanized by CO_2_ inhalation, and the bones with the surrounding soft tissue were aseptically collected. We divided the tibia shaft longitudinally into two parts. One part of the specimens was prepared for scanning electron microscopy (SEM), while the other part was prepared for histological evaluation as previously described. Briefly, the tibias were fixed in 10% neutral buffered formalin, decalcified in 10% EDTA, and embedded in paraffin. The 5-μm sections were Gram stained to assess bacterial colonization. Then, the SEM was performed using a microscope (Inspect Hillsboro, OR, USA) as described above.

### Peptide nucleic acid fluorescent in situ hybridization

After following standard histologic procedures, sections were deparaffinized in xylene and rehydrated by a graded ethanol series (100% × 5 min, 85% × 5 min, 75% × 5 min, and Milli-Q H_2_O × 5 min). One drop of the PNA probe in the hybridization solution, including a FAM-labeled PNA probe (5′-FAM-GAAGCAAGCTTCTCGTCCG-FAM-3′) targeting *S. aureus* 16S rRNA (Servicebio, Wuhan, China), was applied to the rehydrated bone. A coverslip was applied, and the slides were incubated for 90 min at 56 °C. The coverslips were removed, and the slides were incubated for 30 min in the wash buffer (AdvanDx, Woburn, MA, USA) at 56 °C. The slides were removed and dried in the air. The bone sections were covered in approximately 100 μL of 4,6-diamidino-2-phenylindole (DAPI; ThermoFisher, Waltham, MA, USA) and incubated in the dark for 30 min at room temperature. The DAPI solution was washed off with phosphate-buffered saline (PBS), and the samples were allowed to dry in the air. One drop of Gold Antifade Reagent (Thermo Fisher, Waltham, MA, USA) was added to each section. A coverslip was applied, and the samples were incubated overnight in darkness. The coverslips were then sealed with clear nail polish, and the slides were then stored at 4 °C.

### Quantitative real-time polymerase chain reaction

*S. aureus* cells were harvested by scraping from the in vitro biofilm growth. The infected bone and the surrounding soft tissues were also collected for real-time polymerase chain reaction (RT-PCR) assays. Primers were used for amplifying the respective fragments (Additional file 1: Table S1). The total RNA was extracted using a MasterPureTM RNA purification kit (Epicentre) according to the manufacturer’s instructions. Contaminating genomic DNA was removed using Turbo RNase-free DNase I (Ambion). The quantity and purity of the RNAs were analyzed using a Nanodrop 2000 (Thermo Scientific, USA). Then, the mRNA was reverse transcribed to cDNA using the RevertAid First Strand cDNA Synthesis Kit (Thermo Scientific) according to the manufacturer’s instructions. Cyclooxygenase-2 (COX-2), interleukin-6 (IL-6), inducible nitric oxide synthase (iNOS), and tumor necrosis factor-α (TNF-α) were selected as pro-inflammatory indicators. The analysis of the expression of each gene was conducted in triplicate and repeated in three samples. The expression levels of the desired genes were normalized to β-actin as the reference gene and then normalized to the AS*yycG* group using the 2^−△△Ct^ method.

### Statistical analysis

The Bartlett test was performed to assess the homogeneity of data variances, and the Shapiro-Wilk test was conducted to determine the normal distribution of the data. For parametric testing, one-way analysis of variance was used to compare the data, followed by pairwise multiple comparisons with the SPSS software (IBM, Armonk, NY, USA). All data are presented as the mean ± standard deviation. Differences with *P* values < 0.05 were considered statistically significant [22].

## Results

### Antisense *yycG* downregulated the expression of biofilm formation genes and negatively affected the production of the YycG protein in biofilms in vitro

**Fig. 1:**
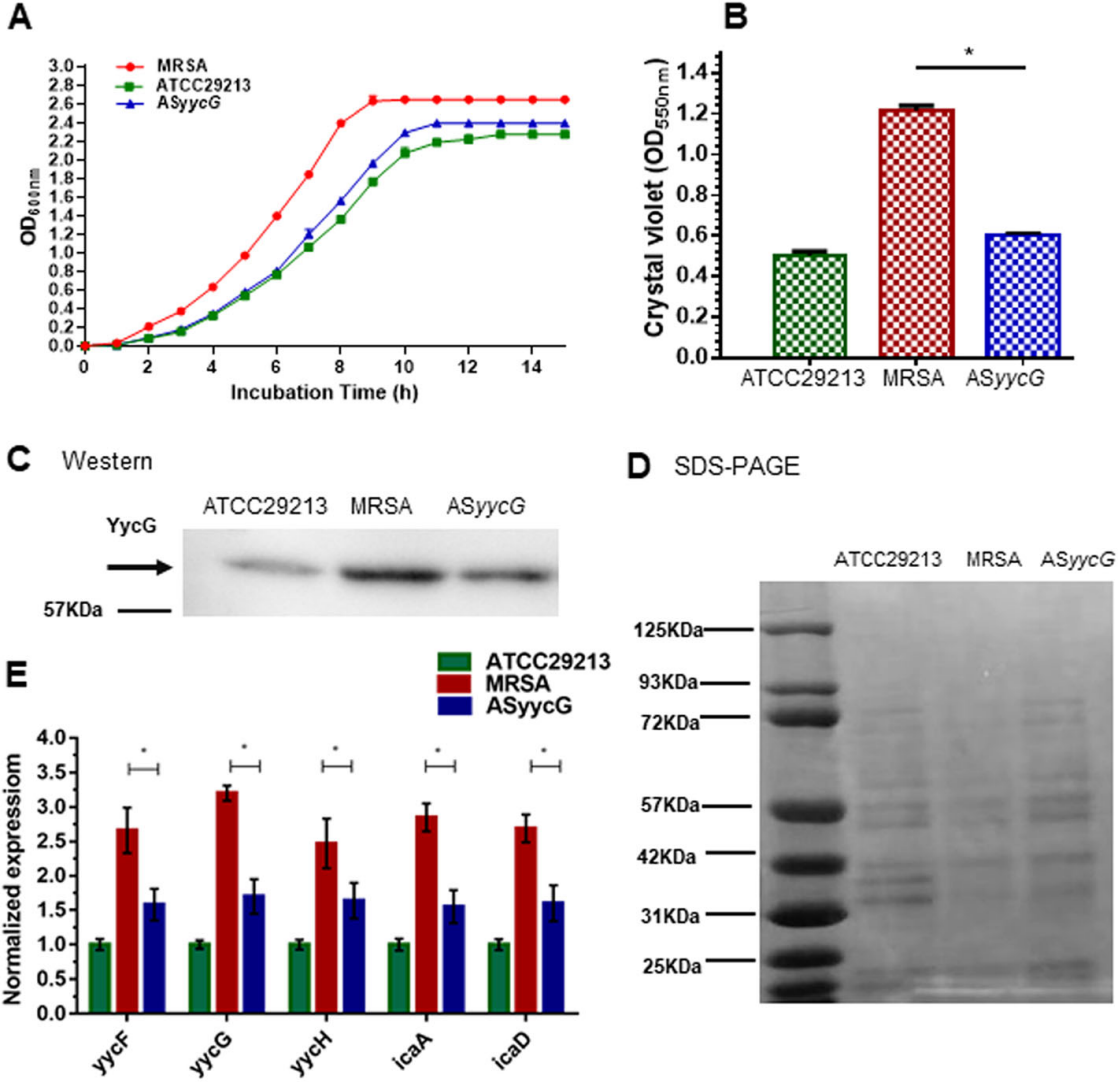
Effects of AS*yycG* on *S. aureus* growth. **a** The growth curves of *S. aureus* strains were compared in three independent experiments. **b** Crystal violet microtiter assay for determining biofilm biomass (*p < 0.05, *n* = 10). **c** YycG production was quantified in Western blots probed with an anti-YycG antibody. **d** Coomassie-stained SDS-PAGE gel showing the equal loading of samples. **e** RT-qPCR measurements were applied for quantifying the expression of biofilm-related genes. Data represent ten biological replicates and are presented as the mean ± standard deviation (*p < 0.05, *n* = 10)

The growth curves of the *S. aureus* strains were compared in three independent experiments (Fig. 1a). AS*yycG* strains biofilm formation was decreased compared to that of the parent MRSA strains (Fig. 1b). Western blotting performed with the anti-YycG antibody showed that the level of the YycG protein was lower in AS*yycG* cells compared with the control cells (Fig. 1c, d), suggesting that the overexpression of AS*yycG* RNA inhibited the translation of *yycG* in *S. aureus*. In general, the AS*yycG* strains showed decreased expression of the genes associated with biofilm formation when compared to MRSA strains during biofilm growth. Comparative quantitative RT-PCR analysis showed that the expression levels of the *icaA*, *icaD*, *yycF*, *yycG*, and *yycH* transcripts were significantly decreased in the AS*yycG* strains compared to the MRSA strains (Fig. 1e).

### Effect of AS*yycG* interference on EPS synthesis and biofilm formation in the bone specimens

**Fig. 2:**
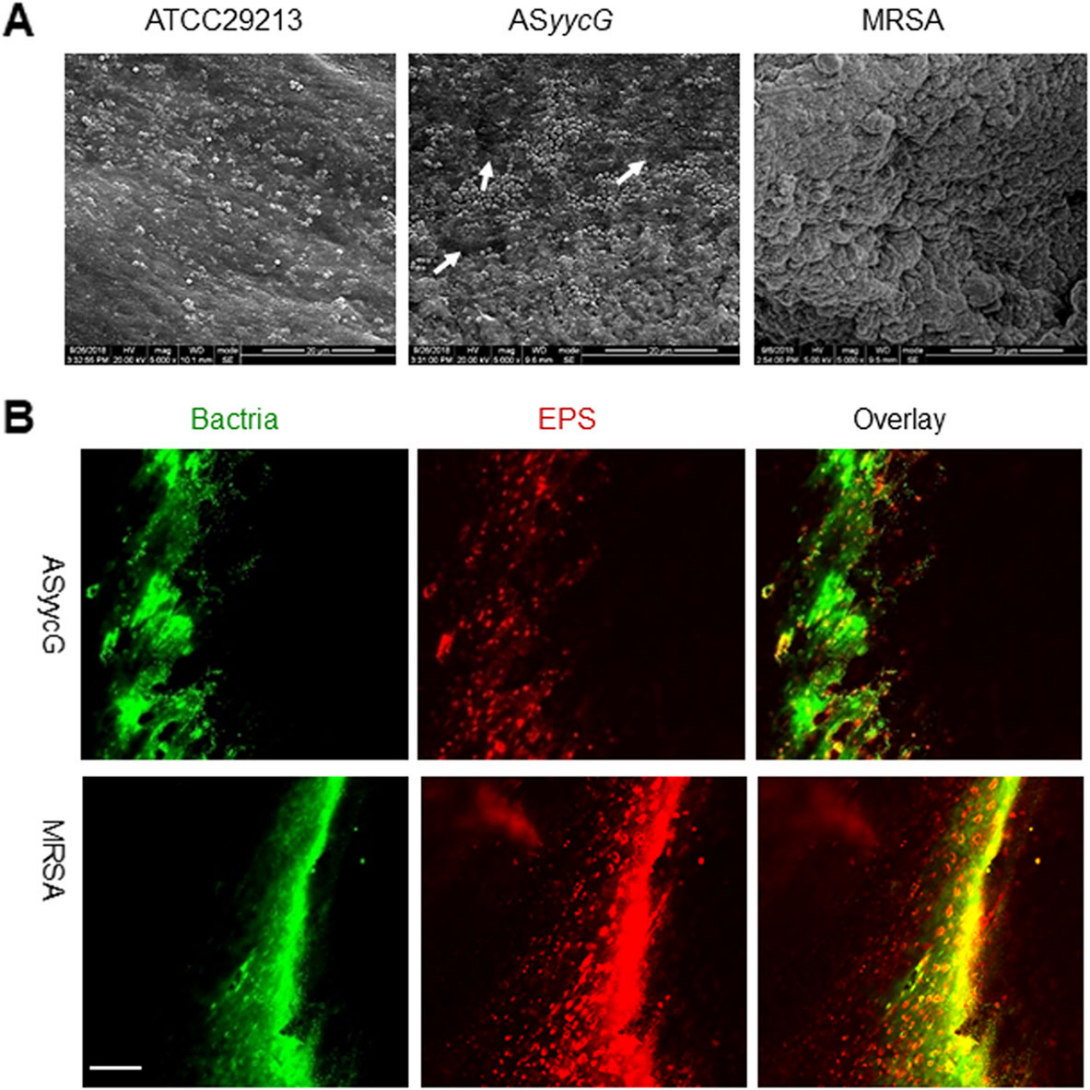
Effect of AS*yycG* interference on EPS synthesis and biofilm formation on bone specimens. **a** SEM of *S. aureus*, AS*yycG*, and MRSA biofilm formation on the bone specimens. **b** Double labeling of the biofilms of the *S. aureus* strains on the bone specimens, green indicates total bacteria (SYTO 9); red indicates EPS (Alexa Fluor 647); scale bars, 100 μm

In addition, the morphology and structure of the bone specimen slides were examined by both CLSM and SEM (Fig. 2). The morphology of the *S. aureus* biofilms was analyzed using SEM. The extracellular matrixes were observed in the MRSA biofilms surrounding clusters of cells, whereas the biofilms of the AS*yycG* group showed little ECM interspersed with “blank” areas (white arrows, Fig. 2a). The double staining of CLSM revealed that silencing of the *yycG* gene markedly decreased the production of EPS, while cells were densely packed within the enriched EPS in the MRSA strain biofilms on the bone specimens (Fig. 2b). These findings were further confirmed by quantitative data revealing that AS*yycG* exhibited a significantly lower EPS/bacteria biomass volume ratio (52.1 ± 5.8%) compared to that of the MRSA strains (76.2 ± 6.6%, *p* < 0.05), indicating a role of the *yycG* gene in EPS architecture development (Fig. 2b). Taken together, the expression of the biofilm-associated genes and the biofilm phenotype were altered in the AS*yycG* strains.

### Micro CT measurement in vivo

**Fig. 3:**
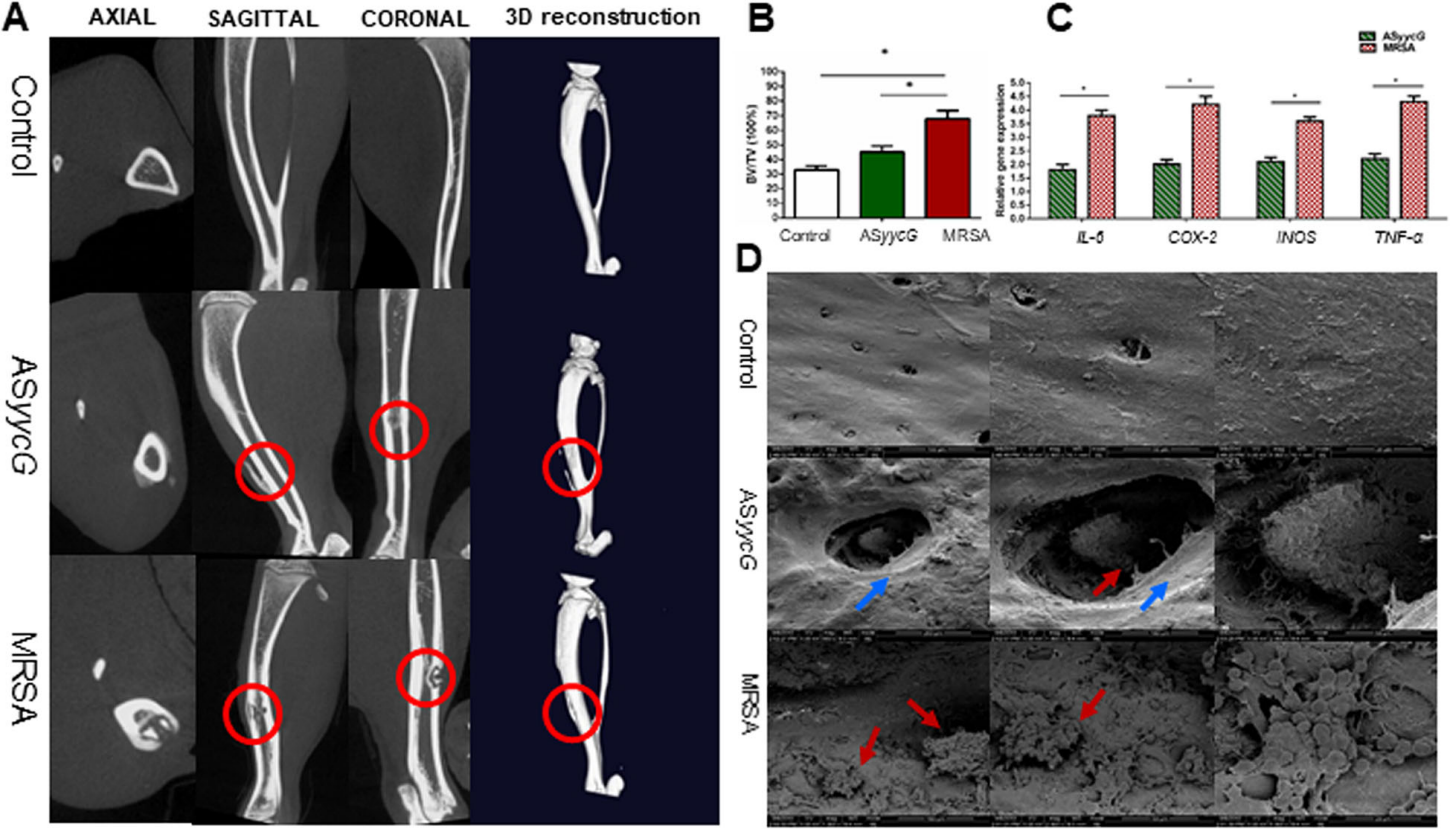
AS*yycG* suppressed the pathogenicity of MRSA in vivo*.* **a** The normal condition showed a clearly defined cortex without apparent signs of osteolysis (upper lane); the osteomyelitis caused by *S. aureus* and the infected regions are indicated (red circle). **b** Average BV/TV values in control, ASyycG and MRSA groups (*p < 0.05, *n* = 10). **c** RT-qPCR for quantifying the expression of inflammatory mediators (*p < 0.05, *n* = 10). **d** Analysis of bone specimens using SEM identified that the spatial organization of microorganisms; The visualized microbial aggregates (red arrows) along the bone samples were generally detected in the peri-drilled sites (blue arrows) surrounding the bone surfaces

The control group showed a clearly defined cortex without apparent signs of osteolysis (Fig. 3a, upper lane). The osteomyelitis caused by MRSA showed critical cortical thickening, loss of cortex integrity, and signs of osteolysis (Fig. 3a, lower lane). Both bone formation and bone resorption were altered around the bone matrix affected by the MRSA strains. AS*yycG* exhibited a thickening of the cortical bone similar to the MRSA group, but osteolysis or destruction was rarely found. Quantitatively, in the control group, the average bone volume (BV)/total volume (TV) values were 33%. The changes were different in the MRSA group, with an average BV/TV value of 68% (*p* < 0.05; Fig. 3b). Surprisingly, the average BV/TV ratio was 45% in the AS*yycG* infected group, which was significantly different than that of the animals infected with the MRSA strains.

### AS*yycG* suppressed invasive ability and pathogenicity in vivo

We next validated the role of AS*yycG* in the invasive ability and pathogenicity of the strain using a rat model of osteomyelitis. The gene expression levels of inflammatory mediators were measured by RT-qPCR (Fig. 3c). In the MRSA group, the mRNA expression levels of IL-6, COX-2, iNOS, and TNF-α were significantly higher than those in the AS*yycG* group at 4 weeks (Fig. 3c)**.** In particular, the expression levels of IL-6 and COX-2 in the MRSA group were two-times higher than those in the AS*yycG* group. These results demonstrated reduced inflammatory reactions in AS*yycG* group compared with the MRSA group. The analysis of the bone samples using SEM was conducted to identify the spatial organization of microorganisms (Fig. 3d). The visualized microbial aggregates along the bone samples demonstrated that microorganisms were generally detected in the periosteum and the structures surrounding the bone surfaces. The aggregated microbes were observed close to the boundary of the periosteum bone surface and the infection sites in the MRSA group (Fig. 3d, lower lane), but the AS*yycG* group samples contained fewer microorganisms (Fig. 3d, middle lane).

**Fig. 4:**
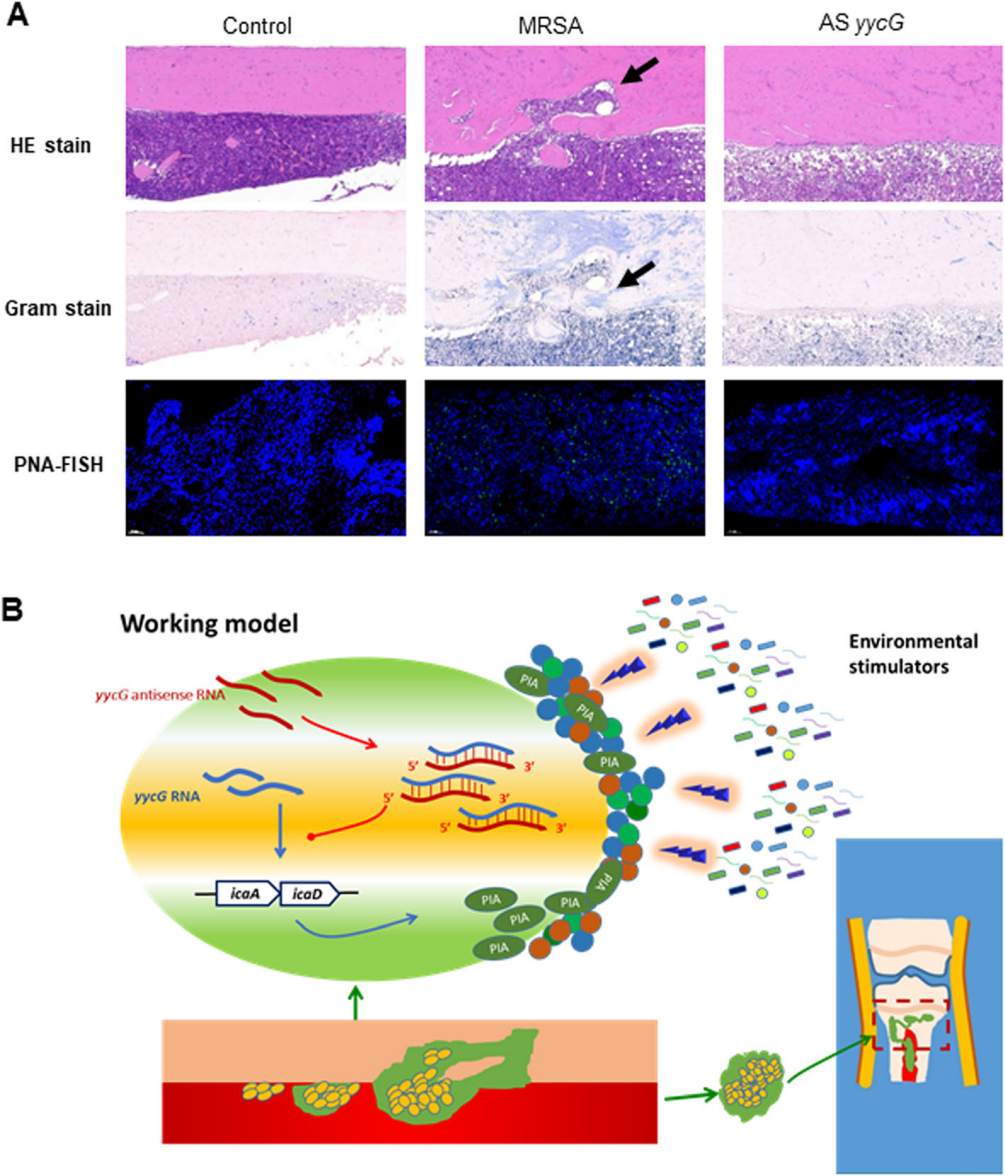
Histological evaluations and peptide nucleic acid fluorescent in situ hybridization. **a** Samples were from the infected and uninfected tibias at 4 weeks. H&E-stained histological sections (upper lane; scale bars: 100 μm). Gram stain samples (middle lane; scale bars: 100 μm). The presence of fluorescent *S. aureus* was identified with a peptide nucleic acid fluorescent in situ hybridization probe for bacterial 16S rRNA (lower lane; scale bars: 100 μm). **b** Working model: AS*yycG* overexpression resulted in the decreased transcription of *icaA* and *icaD*. It was revealed that inactivating the *yycG* gene probably lead to impaired PIA synthesis. The *S. aureus* biofilm suppressed the invasive ability and pathogenicity in AS*yycG* mutants

Histological sections were made from the infected and uninfected tibias at 4 weeks. In the H&E-stained samples (Fig. 4a, upper lane), the thickness of the cortex was increased in the AS*yycG* and MRSA groups. A substantial destruction in the cortex combined with a large amount of inflammatory infiltration was shown in the MRSA group. Gram staining showed that many more microcolonies were contained within the bone of the MRSA group than that of the AS*yycG* group (Fig. 4a, middle lane). After being labeled with a peptide nucleic acid fluorescent in situ hybridization probe for the bacterial 16S rRNA, the presence of fluorescent *S. aureus* was readily identified in the MRSA group (Fig. 4a, lower lane). The fluorescence intensity of the MRSA group was much higher than the AS*yycG* group. This result suggests that AS*yycG* was probably deficient in infection or growth in bone tissues.

## Discussion

Complementary base-pairing to the target mRNA is the major mechanism of antisense RNA that causes the inhibition of the transcription and translation of mRNAs [15]. Here, an AS*yycG* overexpression mutant was constructed, and the YycFG pathway was significantly downregulated in the AS*yycG* strains. As observed by Western blotting, the level of the YycG protein decreased in the AS*yycG* cells, suggesting AS*yycG*-mRNA duplexes could interfere with and inhibit *yycG* gene transcription and translation.

Biofilms represent a complex multicellular community of organisms encased in an extracellular polymeric substance that is composed primarily of exopolysaccharides (EPS) [23]. It has been reported that the EPS matrixes are the crucial components of the protective shelter for antimicrobial resistance in biofilms [24]. Polysaccharide intercellular adhesin (PIA), alternatively termed as poly-β (1-6)-N-acetylglucosamine, is produced by the *ica* operon-encoded enzymes and is the crucial component linked to EPS organization [25]. *IcaA* and *icaD*, as the first two genes of this gene cluster, play a primary role in PIA synthesis. The *icaA* gene encodes a transmembrane N-acetylglucosaminyl transferase that is expressed only in cooperation with the product of the *icaD* gene [26]. RT-PCR assessments revealed that a reduction in the expression of the *ica* operon in AS*yycG* strains may correlate with an inhibited YycFG pathway. Consistently, these altered phenotypes may contribute to the biofilm with impaired EPS accumulation in the AS*yycG* strains. In the current study, the silencing the *yycG* gene by antisense RNA significantly reduced the transcription of the PIA synthesis-associated genes and resulted in a suppressed PIA-mediated biofilm, which likely contributes to impaired EPS deposition at the cell surface in the AS*yycG* strains (working model in Fig. 4b). Thus, these analyses of biofilm formation again confirmed that the YycFG pathway functions in biofilm formation by *S. aureus*.

The bacterial sensitivity to antibiotics and host defense systems are reduced in biofilms, which contributes to the persistence of chronic infections [14]. Using micro-CT scanning in vivo, we reported that the reactive bone formation, bone infarction, and sequestrum establishment were observed surrounding the bone tissues of the rat tibia infected with the MRSA strains. The infectious lesions can migrate out of the intramedullary cavities and extend into the cortex along with Haversian and Volkmann canals, which caused the disruption of cortical blood supply and the sequestrum formation [1]. Nevertheless, AS*yycG* specimens show mainly reactive bone formation with little disruption. This trend indicates AS*yycG* stains exhibited a limited capability to cause infarction of the infected bone tissues. Consistently, the histology evidence from the H&E and Gram staining demonstrated that the capability of the AS*yycG* strains to invade into the hard cortex is reduced compared to that of the MRSA strains. Furthermore, the presence of AS*yycG* bacterial cells in tissues was identified by peptide nucleic acid fluorescent in situ hybridization (PNA-FISH) with CLSM, which explained a higher fluorescence intensity in MRSA group. This result suggests that AS*yycG* was probably deficient in its ability to infect or grow in bone tissues. Taken together, we speculated that AS*yycG* also reduced the pathogenicity and invasiveness of MRSA strains. The overexpression of AS*yycG* will potentially increase the antibacterial efficiency of vancomycin [27].

Proinflammatory mediators and oxidative enzymes will be released by immune cells to participate in the clearing of microorganism colonization, which may result in the destruction of the integrity of cortex barrier [28]. In the present study, we found that the AS*yycG* strains induced lower release of proinflammatory cytokines compared to that of the MRSA strains, and did not induce obvious destruction of the cortex. As previous literature has reported, in a rat osteomyelitis model, the ability to cause tissue damage is associated with the inflammation response, which can significantly promote osteoclast differentiation and activate the RANK/RANKL signaling pathway [29]. *S. aureus* is also known to inhibit osteoblast activity and differentiation. A decrease in proliferation and alkaline phosphatase activity has been shown in vitro models of the *S. aureus*/osteoblast interactions [28]. Taken together, antisense *yycG* RNA plays a role in reducing the inflammatory response compared to the MRSA strains.

## Conclusion

In summary, we identified an antisense RNA with the potential to attenuate the functions of the essential two-component regulatory system element YycG. Furthermore, we showed that the overexpression of AS*yycG* leads to a reduction in biofilm formation and exopolysaccharides (EPS) synthesis compared to the MRSA strains. The AS*yycG* strains exhibited decreased expressions of the *yycF/G/H* and *icaA/D* genes. The AS*yycG* interference inhibited *yycG* transcription and YycG protein production. Furthermore, we demonstrated that ASyycG suppressed the invasive ability and pathogenicity of *S. aureus* in vivo using an SPF rat model. Taken together, the overexpression of AS*yycG* leads to a reduction in the biofilm formation and the bacterial pathogenicity in vivo, which provides a potential target for the management of MRSA-induced osteomyelitis.

## Supplementary information

**Additional file 1:** **Table S1.** Sequences of primers used for qRT-PCR analysis.

## Data Availability

All data generated or analyzed during this study are included in this published article and its supplementary information files.

## Abbreviations

AFM: Atomic force microscopy
CLSM: Confocal laser scanning microscope
CV: Crystal violet
EPS: Extracellular polymeric substances
GO: Graphene oxide
MRSA: Methicillin-resistant *Staphylococcus aureus*
*S. aureus*: *Staphylococcus aureus*
SEM: scanning electron microscopy
