# Supplementary material for: Virulence of methicillin-resistant Staphylococcus aureus modulated by the YycFG two-component pathway in a rat model of osteomyelitis
Source: J Orthop Surg Res. 2019 Dec 12;14:433. doi: 10.1186/s13018-019-1508-z (PMC6909630; doi:10.1186/s13018-019-1508-z)
Supplement: Supplementary file 1 — Additional file 1: Table S1. Sequences of primers used for qRT-PCR analysis. [file 13018_2019_1508_MOESM1_ESM.docx]

**Title Page**

**Virulence of methicillin-resistant *Staphylococcus aureus* modulated by the YycFG two-component pathway in a rat model of osteomyelitis**

***Shizhou Wu ^1^, Yunjie Liu ^2^, Lei Lei^3 *^,*** ***Hui Zhang******^1*^***

^1^ Department of Orthopedics, West China Hospital, Sichuan University, Chengdu, China.

^2^ West China School of Public Health, Sichuan University, Chengdu, China.

^3^ State Key Laboratory of Oral Diseases, Department of Preventive Dentistry, West China Hospital of Stomatology, Sichuan University, Chengdu, China

*** Co-Corresponding author:**

**Lei Lei****,** **PhD,**

Assistant research fellow

State Key Laboratory of Oral Diseases, Department of Preventive Dentistry, West China Hospital of Stomatology, Sichuan University,

NO.14 Renmin South Road, Chengdu City, Sichuan, 610041, China

[leilei@scu.edu.cn](mailto:leilei@scu.edu.cn).

**Hui Zhang, MD, PhD**

Professor

Department of Orthopedics, West China Hospital, Sichuan University, Chengdu, China.

No.37 Guoxue Alley, Chengdu City, Sichuan, 610041, China

[caesarzh@163.com](mailto:caesarzh@163.com)

**Acknowledgments**

We are most grateful to Huiqi Xie for her excellent technical assistance. This study was supported by National Natural Science Foundation of China (NO. 81800964), and Sichuan Provincial Natural Science Foundation of China (NO. 2018SZ0125 and 2019YFS0270).

**Conflict of Interest:**

The authors declare that they have no conflict of interest.

**Ethical standard statement**

All procedures performed in studies involving animals were in accordance with the ethical standards of the institution or practice at which the studies were conducted (Animal Experiments Committee at Sichuan University + No. 2018039A).

**Supplementary materials**

The sequences for recombinant AS*yycG* were blow:

5’GGATCCTACCATAAATCTTCACACGTTGCGTATAGTTACCTCTGGACATTTCGACCGTCTGGTTACG

CATATCGGTGATTGGTTTGGTAATCGTTCGCGCTATAAAGAATCCTAGGATGACTGTGATTAATAATGAAATAGCTGTACCAACAATGAATATTTGATTTATATTATTTAATTGGTTATAAACGTCATTAATTTTTGATTCGATATAAATATTACCAATTACCTTTTTATCGACTTTAACTGGGATATTATATACCCAGACACGGTCCTTACCACCGCCATAATCTTTTAAAATTAAATGATCGTTTGATTGTCCTAGTGATAGTGCTTTTTGGACAGAACTATCATTCGCTTTTTGATTGATTAGACTACGGTTAGACTGCTTCGTCGTCGCAATAATAATTTGGTCTTTATCTATAAAACGAATTTCTCCAATTTCTTGACGGTTGGCATACTCACTTAATAAATTTTGAATATCTTTTTGTGCATTTACGGAGCCCTTTTCGTCATATACTTTTTCAATACTAATTTCTAATTGTTTAGCGTACTGCGTAATATTCTTCTTAAAATTATCAAGCAGCTCTTTTTCAAGGTTATTTGTAAAATACAGCCCGATAATTTGCATACCAATGATAATCAGTAATACATAAACAATTACAAGTTTAGTATGAAGGGATTGTAGTTGTTTTAGCCACTTCATGAATTC 3’

The start sequence of AS *yycG* (700 nucleotides) is underlined. The nucleotides in red indicate the BamHI restriction site and nucleotides in blue indicate EcoRI restriction site.

**Table S1. Sequences of primers used for qRT-PCR analysis**

| **Primers** | **sequence 5’-3’ (Forward/Reverse)** | **Reference** |
| --- | --- | --- |
| **RT-qPCR** |  |  |
| IL-6 | 5’-TGCAATAAGAAGGGCCTGGA -3’/  5’-AGGCTTGCCCCACTACTTAG -3’ | This study |
| COX-2 | 5’- GACGAAATCAACAACCCCGT -3’/  5’- TATTGGCAGAACGACTCGGT -3’ | This study |
| iNOS | 5’ - TCCATCCAGCTGCAAACCTA -3’/  5’ - GCCAGTCTCCTTCCACTCTT - 3’ | This study |
| TNF-α | 5’ - ACACACGAGACGCTGAAGTA -3’/  5’ - GGAACAGTCTGGGAAGCTCT -3’ | This study |
| *icaA* | 5’- GATTATGTAATGTGCTTGGA -3’/  5’- ACTACTGCTGCGTTAATAAT - 3’ | This study |
| *icaD* | 5’- ATGGTCAAGCCCAGACAGAG -3’/  5’- CGTGTTTTCAACATTTAATGCAA -3’ | This study |
| *yycF* | 5’ - TGGCGAAAGAAGACATCA -3’/  5’ – AACCCGTTACAAATCCTG- 3’ | This study |
| *yycG* | 5’ - CGGGGCGTTCAAAAGACTTT -3’/  5’ - TCTGAACCTTTGAACACACGT -3’ | This study |
| *yycH* | 5’ - TCAGTCAGGCGAGCTAACAT -3’/  5’ –CGCTAAGCTTGAACGTACAGA -3’ | This study |
